# Transgenerational Immune Priming in the Field: Maternal Environmental Experience Leads to Differential Immune Transfer to Oocytes in the Marine Annelid *Hediste diversicolor*

**DOI:** 10.3390/genes10120989

**Published:** 2019-12-01

**Authors:** Clémentine Bernier, Céline Boidin-Wichlacz, Aurélie Tasiemski, Nina Hautekèete, François Massol, Virginie Cuvillier-Hot

**Affiliations:** 1 University Lille, CNRS, UMR 8198-Evo-Eco-Paleo, F-59000 Lille, France; clementinebernier29@gmail.com (C.B.); celine.wichlacz@univ-lille.fr (C.B.W.); aurelie.tasiemski@univ-lille.fr (A.T.); nina.hautekeete@univ-lille.fr (N.H.); francois.massol@univ-lille1.fr (F.M.); 2 University Lille, CNRS, Inserm, CHU Lille, Institut Pasteur de Lille, U1019–UMR 8204-CIIL-Center for Infection and Immunity of Lille, F-59000 Lille, France

**Keywords:** TGIP, maternal effect, immunity, hedistin, antimicrobial peptide, lysozyme, MPII, pollutants, Polychaeta

## Abstract

Transgenerational immune priming (TGIP) is an intriguing form of parental care which leads to the plastic adjustment of the progeny’s immunity according to parental immune experience. Such parental effect has been described in several vertebrate and invertebrate taxa. However, very few empirical studies have been conducted from the field, with natural host-parasite systems and real ecological settings, especially in invertebrates. We investigated TGIP in wild populations of the marine annelid *Hediste diversicolor*. Females laid eggs in a mud tube and thus shared the local microbial threats with the first developmental stages, thus meeting expectations for the evolution of TGIP. We evidenced that a maternal bacterial challenge led to the higher antibacterial defense of the produced oocytes, with higher efficiency in the case of Gram-positive bacterial challenge, pointing out a prevalent role of these bacteria in the evolutionary history of TGIP in this species. Underlying mechanisms might involve the antimicrobial peptide hedistin that was detected in the cytoplasm of oocytes and whose mRNAs were selectively stored in higher quantity in mature oocytes, after a maternal immune challenge. Finally, maternal immune transfer was significantly inhibited in females living in polluted areas, suggesting associated costs and the possible trade-off with female’s protection.

## 1. Introduction

Transgenerational immune priming (TGIP) is an intriguing form of parental care that corresponds to a plastic adjustment of the offspring immunity according to parental immune experience. Through TGIP, young are thereby better at fighting a pathogen that previously infected their parents. This immune priming can be highly beneficial to offspring, contributing to their adaptation to the local epidemiological conditions, provided the latter encounter microbial threats similar to their parents [1,2,3]. In invertebrate marine taxa, empirical studies of maternal effects, in general, and of maternal TGIP, in particular, are scarce [4]. Yet, these effects may be of first importance in the response of marine populations to upcoming changes in the dynamics of infectious diseases due to global warming [5]. Theory predicts that, when it occurs, TGIP should most likely benefit the most vulnerable life stages, i.e., the youngest stages, which are usually immunologically not fully competent [6]. In marine species, it often corresponds to gametes, which are most often released in the ocean before fertilization and are thus directly exposed to environmental microbes. Consequently, if TGIP is to evolve in marine invertebrates, it is highly expected to concern gametes, so as to maintain successful fertilization despite unhealthy environment [7].

In vertebrates, the modalities of TGIP especially rely on a maternal transfer of antibodies through yolk or milk, for instance [8]. In invertebrates, which are deprived of such immune effectors, modalities appear more diverse, and underpinning mechanisms often remain elusive [2]. Indeed, many studies have evidenced that an immune challenge of one or both parents enhances the immune performance of the offspring, measured as higher resistance to infection/parasitism or higher immune activity [9,10,11,12,13,14,15,16]. However, in most cases, so many processes occur between the stage of the manipulation (parent immune challenge) and the endpoint measure (immune efficiency in eggs, larvae, or adults produced by the primed parent) that the physiological mechanisms underlying TGIP cannot be properly evidenced. Among possible mechanisms recently uncovered, mothers can directly transfer bacterial constituents to their eggs; this confers no protection to gametes but could serve as immune elicitors in the zygote and developing young [17,18,19]. Mothers may also transfer to their oocytes active immune effectors (e.g., antimicrobial peptides (AMP), proteins with bactericidal properties, pathogen recognition receptors), or their mRNA precursors [15,20,21]. Finally, mothers, as well as fathers, may transfer epigenetic factors (methylation marks, regulatory non-coding RNA) through their gametes, thereby influencing more durably the immunity of their offspring in relation to their own immune experience [3,22,23]. Note that very few of the studies that have so far accumulated knowledge over TGIP modalities in invertebrates have addressed it in wild populations and experimentally investigated TGIP in natural host-parasite systems, which means that many results need field validation [3]. Moreover, the real comprehension of the occurrence of TGIP and its importance in an individual lifetime requires to take into account the ecological settings really experienced. In particular, since TGIP may come at some costs for parents as for offspring, it is highly probable that its occurrence and intensity depend on the local context. In particular, active immune factors transferred to oocytes directly derive from maternal immunity, and thus represent a direct cost to her [24,25,26,27]. Maternal immune investment is thus expected to show some degree of plasticity in relation to the environmental conditions experienced by the mother [28,29]. In some circumstances, she may have to manage trade-offs with other functions and, especially, with her immunity in order to preserve her reproductive value [27].

In the present study, we studied the occurrence and modalities of maternal immune transfer to oocytes in the ragworm *Hediste diversicolor*, a gonochoric species quite tolerant of pollution [30]. Its semelparous reproductive strategy and the female’s habit of laying her eggs in her mud tube, thus sharing microbial threats with the first developmental stages of her progeny, make this worm species a potentially good model to study TGIP. We evidenced an enhancement of the maternal immune protection of oocytes after a maternal immune challenge, in a wild population and the context of a natural host-parasite system. We brought to light immune mechanisms, in the form of maternal transfer of immune components to oocytes, that might contribute to TGIP in this species. We finally compared the extent of such a TGIP between females from different environmental backgrounds, uncovering a possible trade-off between offspring and female’s immune protection.

## 2. Materials and Methods 

### 2.1. Animal Sampling

Worms were collected on three shores chosen for their known differences in terms of sediment contamination. Sampling sites and pollutants’ load of local sediments are extensively described in [31]. Briefly, the site of Authie (AN) is considered as clean and used as a reference site. The site of Boulogne-sur-Mer (shortened here as “Boulogne”, BO) is located inside an industrial harbor and is characterized by sediments contaminated notably by heavy metals and urban and industrial wastes. Finally, the site of Gravelines (GR) is located in an artificial canal that crosses an urbanized area, and sediments there are moderately contaminated compared to Boulogne. We previously demonstrated that the worm populations that live in these three sites shared similar neutral genetic background [31]. For this study, around sixty worms from each site were hand-collected in April 2014 and brought back to the lab together with local water and sediments. Additional sampling of 60 worms was collected in Authie in 2017 for the experiment on the selectivity of the transfer. In all cases, we specifically selected mature females that could be clearly recognized by the greenish color of their tegument [32]. 

### 2.2. Animal Treatment

Bacterial strains used in immune challenges: We chose two bacterial strains that might be plausibly encountered by the worms in their natural habitat: a Gram-positive (gram+) bacteria, *Bacillus hwajinpoensis*, previously isolated by our group from the wall of *H. diversicolor*’s tube sampled in the field (see [31] for details), and a Gram-negative (gram−) bacteria, *Vibrio alginolyticus*, which is an infectious agent widely distributed and responsible of mass-mortality events in marine invertebrates. The injection of heat-killed bacterial solutions at 10^6^ CFU/ml was used to mimic immune challenges. 

Highlighting maternal immune transfer: A total of 4 batches of 15 females from Authie (clean site) were either kept unchallenged (control, C), injected with 10 µl of saline solution (SS), with 10 µl of gram+ bacteria (bacillus), or with 10 µl of gram− bacteria (vibrio). After recovery, worms were kept under controlled conditions for seven days (photoperiod of 10:14; 12 °C for day time and 9 °C for night time; feeding every other day with fish food).

Experiments on local effects: For each geographic origin, 3 batches of 15 females were either kept unchallenged (control, C), injected with 10 µl of saline solution (SS), or injected with 10 µl of gram+ bacteria (bacterial challenge, B). This strain was selected since it induced higher immunoreactivity in *H. diversicolor*. After recovery, worms were kept under controlled conditions for seven days.

### 2.3. Plasma and Oocyte Sampling

The coelomic fluid was collected from individual worms previously anesthetized in filtered seawater (FSW) with 10% ethanol. The samples were kept on ice for 15 min, leading to a clear separation between the dense oocytes, which drop to the bottom of the tube, and the small granulocytes (immune cells), which remain free in the supernatant. The latter fraction was carefully transferred to a new clean tube and centrifuged for 5 min, 10,000 *g* at 4 °C to pellet the cells. The cell-free supernatant was kept as the maternal plasma. The pellet of oocytes was kept for subsequent measures of the antibacterial activity or of mRNA quantity. In the first case, the oocyte pellets were snap-frozen and kept at −80 °C until further processing. In the second case, each oocyte pellet was added with 250 µl of Qiazol (Qiagen, Hilden, Germany), then snap-frozen and kept at −80 °C until further processing. When necessary, a small number of oocytes were retrieved from the sample before processing to measure the mean diameter of the oocytes with a stereomicroscope Motic (Motic SMZ 168) and the Images plus 2.0 software (30 oocytes randomly chosen per sample). 

### 2.4. The Measure of the Antibacterial Activities in Plasma and Oocytes

The antibacterial activities of plasma and oocyte samples were assessed in 10 µl of each sample, using the antibacterial liquid growth assay described in [31]. This assay provided an antibacterial score for each sample, obtained as the log_2_ of the largest dilution at which antibacterial activity was detected. The higher the score, the higher the ability to kill the tested bacteria. Results are given as a distribution of the antibacterial scores obtained for each studied condition. 

### 2.5. Detection of Hedistin Peptide and mRNA in Oocyte Samples

Hedistin is an antimicrobial peptide (AMP) specifically produced by *Hediste diversicolor*. Tasiemski and co-workers evidenced that Natural killer (NK) cells-like constitutively expressed the hedistin gene and released the active peptide in the surrounding cœlomic liquid upon bacterial challenge. Active hedistin shows clear antibacterial activity against a broad spectrum of gram+ bacteria, and also against the gram− bacteria *Vibrio alginolyticus* but with less efficiency [33]. Note that hedistin gene expression produces an inactive peptide named preprohedistin and that several post-translational cleavages and modifications are needed to release the active hedistin peptide. 

To detect hedistin peptide in oocyte samples, we carried out a dot-blot experiment with an antibody that was specifically raised against the almost complete sequence of hedistin. The chemically synthesized immunogenic sequence (LGAWLAGKVAGTVATYAWNRYV) was coupled to ovalbumin and used for the immunization procedure of a rabbit (COVALAB, Villeurbanne, France). The hedistin antiserum obtained thus detected the active peptide but also the inactive forms of the peptide. 

The oocytes of 12 female worms from Authie were collected after a similar process, as explained above. For each condition considered (control, saline, bacillus, and vibrio), three females were assayed. The raw quantity of proteins per sample was quantified by Bradford assay (Bradford Reagent B6916, Sigma-Aldrich, St Louis, MO, USA), then 5 µg of total proteins per sample was adjusted in 1.5 µl of sterile water and plotted onto the nitrocellulose membrane (BIO-RAD, Hercules, CA, USA). After incubation in blocking buffer (1 h; TBS, 0.1 M–Tween 20, 0.05%–non–fat dry milk, 2%), the membrane was probed with the rabbit polyclonal anti-hedistin antibody (2 h, 1/500 dilution), washed 3 times (TBS, 0.1 M–Tween 20, 0.05%), and then incubated with the peroxidase-conjugated anti-rabbit secondary antibody (Abcam, Cambridge, UK; 1/5000; 2 h in TBS-Tween 20, 0.05%). A ClarityTM Western ECL Substrate (BIO-RAD) was used for the chemoluminescence visualization of the immunolabeling with a Kodak Bio Max light film. 

To assay the histological distribution of preprohedistin mRNA in females, in situ hybridization was performed on paraffin-embedded sections of the whole body of unchallenged females. Probes used and procedures were similar to those described in [33].

### 2.6. Quantification of mRNA Levels in Oocyte Samples

Total RNAs were extracted from oocyte samples (*n* = 4 per site and condition) according to Qiazol manufacturer’s instructions. Genomic DNA was retrieved by incubation with DNAse RQ1 (Promega, Madison, WI, USA) according to the manufacturer’s instructions. Reverse transcription was performed on 1 µg of total RNAs with the RevertAid kit (Thermofisher Scientific, Waltham, MA USA) using a mix of OligodT (500 ng/µl) and random primers (250 ng/µl). The cDNA obtained was then used to measure by qPCR the relative quantity of mRNA of several genes of interest compared to that of GAPDH used as a reference gene. Three genes were investigated: hedistin—an AMP; lysozyme—an enzyme with antibactericidal activities; and MPII—a metalloprotease involved in the detoxification of heavy metals and proved to display antibacterial activity [34]. The sequences of the couples of primers used and their efficacy were the following: hedistin (F: GATGCAAAGAGGGTGGAAGA; R: TCGATTCCACGCGTATGTAG), E = 2.01; lysozyme (F: CCGTATCAGATCAAGGCAATC; R: GATTGGAGCGGTATTTCCAG), E = 2; MPII (F: AGGAAACAACGCTGACAACC; R: GCTTCTTCTTGTGGGAATCG), E = 1.98; GAPDH (F: CGTATTGGACGTCTGGTCCT; R: TAATCGGCTCCAACAGATCC), E = 1.99. Reactions were run on a LightCycler 480 (Roche, Basel, Switzerland), using iTaq Universal SYBR Green Supermix (Bio-Rad, Hercules, CA, USA) with the following cycling conditions: 95 °C for 30 s (1 cycle), 95 °C for 15 s, and 59 °C for 45 s (40 cycles). A single fluorescence read was taken at the end of each 59 °C step, and a sample was considered positive if the Cq value was less than 35 cycles. Ratios of expression between each gene of interest and GAPDH were calculated using the second derivative method with the Roche LightCycler 480 software (v 1.5.1).

### 2.7. Statistical Analyses

Mean oocyte diameters and distributions of antibacterial scores between treatments were compared through Kruskal–Wallis tests followed by pairwise comparison of Dunn, using the Benjamini–Hochberg adjustment for multiple comparisons to assess which groups were dissimilar from one another (“dunn.test” R package, [35]). For anti-bacillus and anti-vibrio scores measured in the same oocyte samples and for anti-bacillus scores measured in oocyte and plasma collected from the same females, statistical comparisons were performed through exact Wilcoxon signed-rank tests for paired data (“Wilcox.exact” function in exactRankTests R package). 

To compare gene expression ratios and because we had small sample sizes (*n* = 4 per site and condition), we used permutational analyses using the “lmp” function from the lmPerm package in R. Lmp function returns type III sums of squares and implements analysis of variance models, but calculates permutation probabilities, making no assumptions about underlying distributions. After permutational analyses of variance testing for treatment and site effects and their possible interaction, Tukey’s HSD post hoc tests were carried out to infer pairwise differences in gene expression between sites and treatments. Global models tested and detailed output of Tukey tests are given in Appendix A.

All the datasets produced by the present study are provided as Supplementary Materials.

## 3. Results

### 3.1. Antibacterial Activities of Plasma and Oocytes

To be sure to compare similar items, we first checked that the mean diameter of the oocytes produced by females according to their treatment (control, saline-injected, bacteria-injected) was similar in each worm population (Kruskal–Wallis tests, *p* > 0.05). A given volume of oocytes homogenate thus corresponded to a similar number of cells for each treatment.

**Figure 1:**
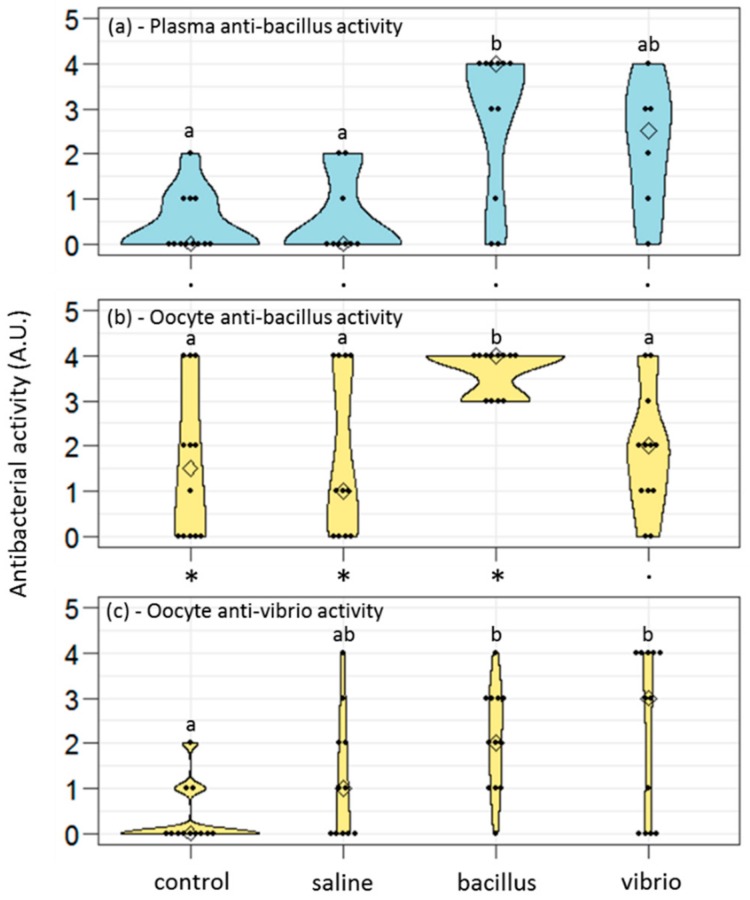
Levels of antibacterial activities in the plasma (**a**) and the oocytes (**b**,**c**) of female worms challenged or not by bacterial injection. Females may be naive (“control”), sham-injected with a saline solution (“saline”), or challenged with heat-killed bacteria of the gram+ strain *Bacillus hwajinpoensis* (“bacillus”) or of the gram− strain *Vibrio alginolyticus* (“vibrio”). For each female, oocyte samples had their antibacterial activity estimated against bacillus (**b**) and vibrio (**c**). For b and c: control, *n* = 12; saline, *n* = 11; bacillus, *n* = 12; vibrio, *n* = 12. When in the sufficient quantity, the plasma from the mother also had its antibacterial activity estimated against bacillus (a: control, *n* = 12; saline, *n* = 9; bacillus, *n* = 11; vibrio, *n* = 6). Within the violin plots, the diamond spot is the marker for the median, and the black dots represent raw data. The thickening of the colored plot shows the probability density of the data at different values (kernel density estimation). Different letters point out significantly different scores between treatments in Dunn’s tests (horizontal comparisons). An asterisk rather than a point indicates significantly different scores between paired data (vertical comparisons: plasma vs. oocyte anti-bacillus activities or oocyte anti-bacillus vs. anti-vibrio activities).

When injected with bacillus, females showed a clear increase in the anti-bacillus activity of their plasma compared to control or saline-injected females (Figure 1a; Table 1). However, females injected with vibrio, also showed an increase of the anti-bacillus activity of their plasma, although less important. It means that the immune reaction of females, as measurable 7 days after the challenge, showed low specificity since females challenged with a gram+ bacteria had similar quantities of anti-gram+ immune effectors in their plasma as females challenged with a gram− bacteria (Table 2).

By contrast with plasma, the oocytes of gravid females challenged by bacillus showed significantly higher anti-bacillus activity than oocytes of gravid females challenged by vibrio (Figure 1b; Table 1 and Table 2). Females that encountered a gram+ bacteria thus enhanced the immune defense of their oocytes selectively against gram+ bacteria. The anti-vibrio response appeared different: females challenged with vibrio might produce oocytes with a higher content of anti-vibrio immune effectors, but not more than females challenged with bacillus, or even from females injected with saline solution (Figure 1c; Table 1 and Table 2). Accordingly, when comparing the antibacterial scores (anti-bacillus and anti-vibrio) from individual females, we observed that females challenged with bacillus produced oocytes with higher anti-bacillus than anti-vibrio activity (Figure 1b,c; exact Wilcoxon signed-rank test for paired data, *p* = 0.002), while females challenged with vibrio produced oocytes with similar anti-bacillus and anti-vibrio activities (*p* = 0.36). TGIP thus seemed more efficient towards gram+ bacteria than a gram− bacteria in this species. Interestingly, control females and saline-injected females also provided higher anti-bacillus than anti-vibrio immune effectors to their oocytes (control: *p* = 0.02; saline: *p* = 0.03). On the other hand, no differences were detected between the anti-bacillus activities of the oocytes and plasma whatever the treatment of the female (Figure 1a,b; exact Wilcoxon signed-rank test for paired data, control, *p* = 0.054; saline, *p* = 0.19; bacillus, *p* = 1; vibrio, *p* = 0.13). 

Overall, a recent infection of the mother translated into a higher load of antibacterial materials stored in the oocytes. However, the specificity of the immune storage into the oocytes appeared higher for anti-gram+ defense than for anti-gram− defense. In other words, bacterial infection and, to a lesser extent, injury (saline injection) of the mother led to some anti-gram− immune effectors to be stored in the oocytes; by contrast, some anti-gram+ immune effectors were stored in the oocytes even in control condition, and females specifically enhanced this stock after a challenge with a gram+ bacteria.

### 3.2. Hedistin Peptide and mRNA in the Maturing Oocyte

**Figure 2:**
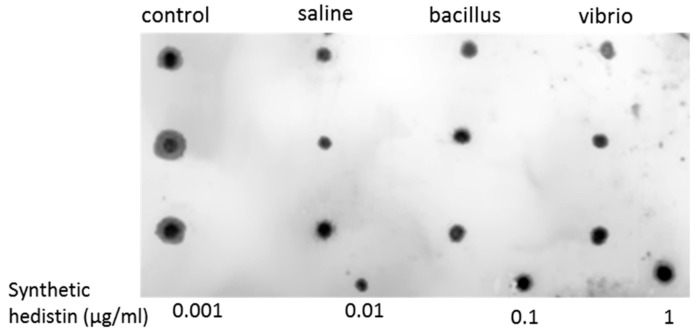
Immunodetection of hedistin in oocytes of female worms challenged or not by bacterial injection. Females might be naive (“control”), sham-injected with a saline solution (“saline”), or challenged with the gram+ strain *Bacillus hwajinpoensis* (“bacillus”) or the gram− strain *Vibrio alginolyticus* (“vibrio”). For each condition, three females were sampled, and the equivalent quantity of their oocyte sample was deposited on the membrane in vertical lines. The presence of hedistin (and its precursors) in the samples was detected by an anti-hedistin antibody. Larger and darker dots indicate more hedistin (and precursors) in the sample. As a standard, a dilution range of synthetic hedistin was plotted at the bottom of the membrane.

To investigate the relative role of hedistin in the antibacterial activities measured in oocytes, we assessed the presence of the peptide in oocyte samples by dot blot. Hedistin peptides targeted by the antibody (active hedistin + prohedistin + preprohedistin) were detected in the oocytes of females of all conditions (control, saline-injected, bacillus-injected, and vibrio-injected; Figure 2), suggesting that the antibacterial activities measured could be at least partly due to the storage of this AMP. However, measuring and comparing the size and intensity of the dots with ImageJ gave no significant differences between the four treatments (KW chi-squared = 6.08, df = 3, *p* = 0.11).

**Figure 3:**
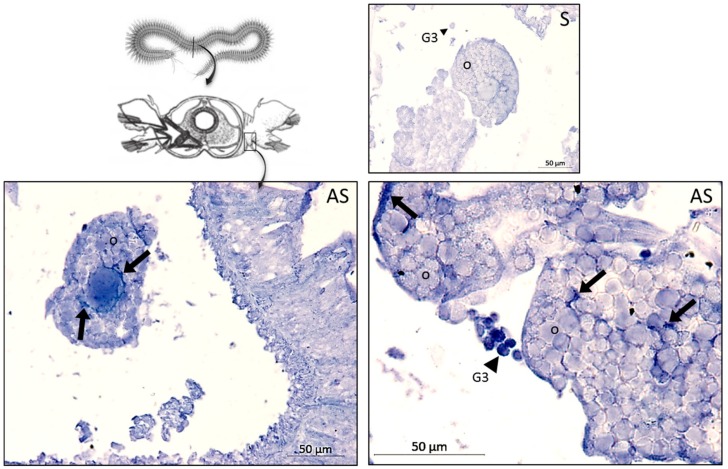
Storage sites of hedistin mRNA in unchallenged females. Transversal slices of naive *Hediste diversicolor* female hybridized with an antisense probe (AS) or with a control sense probe (S) that targets the preprohedistin mRNA. The producing granulocytes G3 are specifically labeled in antisense conditions (AS, arrowheads), as noticeable by the dark blue labeling of the cell content, together with the cytoplasm of the growing oocytes (o) that float freely in the cœlomic cavity (arrows). Round shapes inside oocytes correspond to yolk platelets. Scheme adapted from BIODIDAC© Jon Houseman.

In addition to the peptidic forms of hedistin—already active or quickly turned active by enzymatic cleavage of prohedistin and preprohedistin—we evidenced the presence of other precursors of hedistin in the oocytes in the form of mRNAs. FISH labeling with an anti-sense probe confirmed the presence of a high quantity of hedistin mRNAs in immune G3 cells (arrowheads, Figure 3), but also revealed the storage of hedistin mRNAs in the cytoplasm of the oocytes (arrows, Figure 3). 

**Figure 4:**
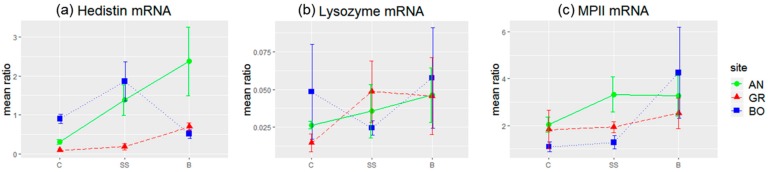
Relative levels of hedistin (**a**), lysozyme (**b**), and MPII (metalloprotease II) mRNA (**c**) in the oocytes of females from Authie (AN), Gravelines (GR), and Boulogne (BO) after no treatment (C), saline injection (SS), or bacterial injection (B). Authie is a reference site considered as clean; Boulogne and Gravelines are polluted shores. Gene expression, measured by RT-qPCR, was estimated by the ratio between the specific gene mRNA level and the GAPDH mRNA level used as an internal reference. The bacterial injection was made with the *Bacillus hwajinpoensis* strain. Results are given as the mean of four independent measures ± sem for each site and condition.

We quantified the relative proportion of hedistin mRNAs in oocytes of females collected in Authie (clean site) and submitted or not to an immune challenge (bacillus injection). The relative quantity of hedistin mRNAs in oocytes was higher for mothers saline-injected or challenged by a bacillus (Figure 4a, AN plot). Permutational analyses of variance detected a significant effect of the treatment on the hedistin mRNA ratio (Table 3). Moreover, the bacteria-injected group from AN stood apart from the control group (Tukey’s HSD post hoc tests, diff = 2.07, *p* = 0.01), whereas the saline-injected group had an in-between response, being statistically different neither from the control group (*p* = 0.51) nor from the bacteria-injected one (*p* = 0.61). A load of hedistin mRNAs in oocytes thus specifically increased in response to a bacterial challenge of the mother by an environmental bacterial strain sensitive to hedistin (here, the gram+ *B. hwajinpoensis*). As a comparison, lysozyme and MPII mRNAs were statistically not more abundant in oocytes from injected females (Figure 4b,c, AN plot; see Table 3 and Appendix A for statistical details).

### 3.3. Influence of the Maternal Environment on Immune Transfer to Oocytes

**Figure 5:**
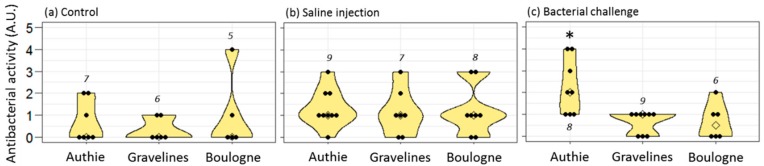
Levels of antibacterial (anti-bacillus) activities in the oocytes of female worms from three geographical origins, challenged or not by bacterial injection. Authie is a reference site considered as clean; Boulogne and Gravelines are polluted shores. For worms from each site, females might be naive (**a**), sham-injected with a saline solution (**b**), or challenged with heat-killed bacteria of the gram+ strain *Bacillus hwajinpoensis* (**c**). Within the violin plots, the diamond spot is the marker for the median, and the black dots represent raw data. The thickening of the colored plot shows the probability density of the data at different values (kernel density estimation). Numbers indicate sample size, and an asterisk points out significantly different scores.

Contrarily to Authie, Boulogne and Gravelines have sediments contaminated with heavy metals and urban/industrial wastes, more heavily in Boulogne than in Gravelines [31]. Comparing oocytes of females from these three sites, we observed no difference in oocyte antibacterial activity for control females (Figure 5a; KW chi-squared = 0.41, df = 2, *p* = 0.81) and saline-injected females (Figure 5b; KW chi-squared = 0.31, df = 2, *p* = 0.86). By contrast, females challenged by bacillus showed different oocyte antibacterial activity depending on their geographical origin (Figure 5c; KW chi-squared = 9.26, df = 2, *p* = 0.01). Clearly, only females from Authie stored higher quantity of anti-bacillus immune effectors in their oocytes after an immune challenge by bacillus (Dunn’s tests: Authie vs. Gravelines, *z* = 2.71, adj *p* = 0.01; Authie vs. Boulogne, *z* = 2.51, adj *p* = 0.009; Gravelines vs. Boulogne, *z* = −0.07, adj *p* = 0.47).

The specific accumulation of hedistin mRNA in oocytes of immune-challenged females also appeared restricted to females from Authie (Figure 4a). Indeed, contrarily to females from Authie, females from Gravelines or Boulogne had similar loads of hedistin mRNA in their oocytes whatever the treatment and did not accumulate hedistin mRNAs after an immune challenge ((BO: C / SS / B) or (GR: C / SS / B): all combinations non-significant in Tukey’s HSD post hoc tests; see Appendix A for statistical details). Consequently, bacteria-injected females from Authie presented higher loads of hedistin mRNA in their oocytes compared to females from Boulogne (Tukey’s HSD post hoc tests, diff = 1.87, *p* = 0.03) and to a lesser extent from Gravelines (diff = 1.68, *p* = 0.07). Moreover, permutational analyses of variance evidenced a clear site effect for the hedistin mRNA ratio (Table 3). More precisely, females from Gravelines had oocytes with globally lower loads of hedistin mRNA compared to females from Authie (Tukey’s HSD post hoc tests, diff = −1.03, *p* = 0.005) and also Boulogne (diff = −0.77, *p* = 0.04). An interaction between the site and treatment was also evidenced (Table 3). It mainly concerned females from Boulogne compared to that from Authie, with no difference for control females (Tukey’s HSD post hoc tests, diff = 0.59, *p* = 0.9) or saline-injected females (diff = 0.48, *p* = 0.9) but almost twice more hedistin mRNAs loaded in oocytes of bacteria-injected females from Authie (diff = 1.87, *p* = 0.03). As a whole, females from Authie stored more hedistin mRNA in their oocytes, moderately after saline injection and clearly after bacterial challenge, whereas females from both polluted sites appeared unable to make such an immune transfer. Females from Boulogne were seemingly less affected, but only for saline-injected females.

No differences between sites of origin were detected concerning lysozyme mRNA load (Figure 4b, see Table 3 and Appendix A for statistical details). 

Finally, permutational analyses of variance evidenced a treatment effect for MPII mRNAs (Figure 4c, Table 3). However, none of the pairwise differences were significant (see Appendix A for statistical details). We could thus conclude a small accumulation of MPII mRNA in oocytes after injection, which seems mainly due to the females from Boulogne that might tend to enhance MPII loading after bacterial challenge (Tukey’s HSD post hoc tests, diff = −1.70, *p* = 0.052). All the datasets produced by the present study are provided as Supplementary Materials.

## 4. Discussion

The present study evidenced a maternal transfer of immunity in the annelid Polychaeta *Hediste diversicolor*, a semelparous coastal worm described as tolerant to pollution. We provided here evidence that females’ immune experience specifically led to the production of oocytes with a higher load of antibacterial defense and of mRNAs coding for antibacterial effectors. Interestingly, anti-gram+ and anti-gram− responses did not seem equivalent, the former leading to a more effective phenomenon of transgenerational immunity. This maternal immune transfer was inhibited in females living in polluted areas. We uncovered here possible costs and trade-offs associated with this maternal protection.

To date, TGIP has been reported in all major invertebrate phyla (insects [10,11,12,21,36,37], crustaceans [1,38,39], molluscs [20,40]), except annelids. The present study filled this gap by evidencing TGIP in a marine polychaete. Our study also documented, for the first time, a TGIP occurrence in a semelparous species. Zanchi et al. previously suggested that TGIP might be restricted to iteroparous species because of the cost it represents for the female [16]. Our results showed, on the contrary, that iteroparity was not a prerequisite to the occurrence of TGIP, and suggested that this costly maternal investment could evolve if it comes along with a whole set of life-history traits that render the benefits of TGIP sufficient to balance the associated costs [2]. In the present case, some peculiarity of the life cycle of the species might have promoted TGIP evolution. As an exception among the Nereididae, *H. diversicolor* is characterized by the absence of a planktonic larval stage [41]. Its lifecycle is rather holobenthic, with the eggs laid by females in her mud-tube and fertilized in situ by males. Larvae then fulfill their development into the female’s tube, all the while protected by their mother that will die soon after the larvae have quit the tube [42]. As a consequence, mothers and young stages (gametes, embryos, young larvae) share the same environmental microbiota of the mud-tube. Maternal exposure to pathogens constitutes thus a good predictor of the likelihood of offspring exposure, which may have promoted the evolution of TGIP as a form of transgenerational adaptive plasticity [43]. Comparisons with genetically close species that produce dispersing larvae (e.g., *Platynereis dumerilii*) would help corroborate the importance of the predictability of the offspring environment in the evolution of TGIP in invertebrates [44,45].

Specificity refers to the degree to which the immune system is able to differentiate immune elicitors (here bacteria). In invertebrates, the immune response is not as specific as in the vertebrates that evolved effectors of very high specificity towards their target (i.e., antibody or lymphocyte receptors). However, some degree of specificity, as the ability to discriminate large families of pathogens, has been described in many models [46]. In our experiments, females challenged by gram+ or gram− bacteria did not respond equivalently in terms of TGIP. A gram+ challenge led to selective transgenerational priming, meaning that oocytes from mothers challenged by gram+ bacteria had a clear higher defense against gram+ than against gram− bacteria. By contrast, mothers challenged by gram− bacteria transferred equivalent anti-gram+ and anti-gram− defenses to their oocytes. It implied that the immune system of females was able to discriminate between the two bacterial threats and to respond to them differently, loading different quantities and/or type of antimicrobial effectors in their growing oocytes. This quite specific detection of pathogens has been suggested to rely on a diverse and complex innate immune sensing system in invertebrates [47]. Some degree of specificity in TGIP had already been described in other models, such as the insects *Tribolium castaneaum* [48], *Tenebrio molitor* [49], or the crustacean *Daphnia magna* [1]. In the case of *Hediste*, further investigations, especially with many more strains of pathogens, are needed to correctly evaluate the degree of specificity of the sensing system.

Interestingly, we observed that control females protected their future eggs with basal levels of anti-gram+ molecules but virtually none anti-gram− effectors. They did so while they have very few anti-gram+ molecules in their plasma, suggesting an active accumulation of these molecules into the oocytes, rather than a passive impregnation of the oocytes from the maternal plasma. Also, saline-injected females produced oocytes with low levels of anti-gram+ defenses but higher than anti-gram− defenses. In all, investment in anti-gram+ protection appeared favored against anti-gram− protection. This might be linked to the nature of the bacterial threat: gram+ bacteria are not abundant in seawater (<5%) but appear far more present in marine sediments [50], following the example of *Bacillus hwajinpoensis* that was isolated from the wall of *Hediste*’s mud tube [31]. In this case, the worms daily coexist with such strains and need permanent constitutive immune processes to control bacteria over-proliferation and subsequent risk of invasion, as already suggested in other invertebrate models [51,52]. On the contrary, opportunistic infections, as those provoked by *Vibrio alginolyticus,* are rather fought by inducible mechanisms [53]. Thus, the higher probability of encountering gram+ bacteria might have favored the evolution of more efficient mechanisms for the detection and transfer of anti-gram+ effectors, as similarly suggested in the insect *T. molitor* [25,49]. 

We evidenced in the present study that oocytes of primed females showed higher antibacterial activities compared to naive females, suggesting that within a short period (7 days here), the oocytes’ content in antimicrobial molecules rose, thus providing readily active protection to the gametes that would be released in the environment. This protection was possibly due, at least in part, to the storage of active hedistin, an AMP with clear anti-gram+ activity, into the oocytes. The dot blot experiment proved that hedistin was present in oocytes, but this technique could not distinguish between readily active form (hedistin *per se*) and inactive peptidic precursors (prohedistin and preprohedistin, which need to be enzymatically cleaved to produce active hedistin). The two forms might be stored in the gametes, conferring two distinct levels of protection: the mature peptide provides immediate protection as it is readily active; the (pre)propeptide is inactive but more stable and can thus constitute somewhat longer-term protection. In our case, the high content of the whole hedistin in oocytes of control females might result from a high content of inactive pro- and preprohedistin but few quantities of active hedistin, which would translate into low antibacterial activity, explaining the inconsistency between dot blot and antibacterial tests results. The ratio might change in oocytes from challenged females via the conversion from inactive to active hedistin in response to the maternal immune challenge. We argued that such a mechanism of enzymatic activation of immune effectors is a possible newly evidenced mechanism of TGIP. However, more specific dosages would be necessary to conclude the specific accumulation of the different forms of hedistin in oocytes following a maternal challenge. 

In the same line, we evidenced that hedistin-encoding mRNAs were specifically stored in the oocytes after a bacterial challenge of the mother. This storage did not result from a general increase in the concentration of immune genes mRNAs in oocytes, as proved by the constant load of lysozyme mRNAs, whatever the treatment. Accumulation of maternal mRNAs in oocytes during oogenesis is a phenomenon largely described throughout animal kingdom [54]. These mRNAs—either synthesized by the oocyte itself or acquired from its neighborhood (e.g., transfer from nurse cells)—appear remarkably stable (long half-lives) and translationally quiescent (not translated into proteins) during oogenesis. Their function is to compensate for the absence of transcription in mature oocytes and early zygotes, the genome of which remains silent until the maternal-to-zygotic transition is completed (MZT, also referred to as mid-blastula transition) [55]. In the closely related annelid *Platynereis dumerilii*, the MZT has been estimated to occur at 8–10 h post-fertilization (end of segmentation, beginning of cell differentiation processes, [56]). Until this point, the embryo is as exposed and intrinsically undefended as mature gametes, as it is unable to produce its protective molecules from its genome. By storing hedistin mRNAs in their gametes, females thus potentially confer protection to the future zygotes and embryos, specifically against pathogens they have a good chance to encounter. Through such delayed mechanisms, the influence of the mother’s environment on her offspring immunity extends over a period that clearly exceeds the lifetime of proteins she could synthesize and transfer. A thorough study, in the basal metazoan *Hydra vulgaris*, likewise dissected the dynamics of a maternal transfer of mRNAs coding an AMP, perculin1a. In this model, the maternally derived AMP helped control and shape the symbiotic microbiota that colonized the budding individual [57]. Our results on hedistin protein and mRNA thus made this AMP a good candidate to be involved in the short-term (as protein and pre-protein) and/or long-term (as mRNA) protection of offspring in *H. diversicolor*. However, it is possible that immune mechanisms other than hedistin transfer also contribute to TGIP in this species, notably via the transfer of other antibacterial molecules not investigated yet. 

Since immunity is costly, a trade-off between the female’s immunity and the one she transfers to her progeny is expected and has been evidenced in other invertebrate models [26,58]. This cost could be manifested as reduced fecundity, egg size, or hatching rate. It may also translate into subtler effects on females’ survival rate due to negative effects on females’ immunity. No direct cost on egg size was evidenced here. By contrast, we revealed a possible hidden cost on females’ survival rate by comparing several worm populations exposed to additional environmental stressors. *Hediste* worms live into a tube dug into the mud; they are thus directly exposed to the pollutants mixed to the sediments, leading to possible adverse health outcomes. In this respect, we previously demonstrated that *Hediste* worms that live in the polluted sites of Boulogne and Gravelines had no cost on reproduction (no reduction in fecundity) but suffered from several immune disturbances responsible of lower immune resistance in front of local pathogens [31]. In the present study, we further evidenced that females from the polluted sites were also unable to protect their progeny by immune transfer following an immune challenge. This concerned both active antibacterial molecules and hedistin mRNAs. In this context, we hypothesized that females from Authie did pay a cost to TGIP and that this cost could not be borne by females from Boulogne and Gravelines that already suffered from personal immune costs due to their toxic environment. Here, the need for females to survive infections is an absolute priority because of their semelparous mode of reproduction: they must survive until the end of their unique gametic maturation to expect any fitness gain. It is thus of no surprise that females already weakened by their noxious environment should favor their immunity to that of their eggs when facing an immune threat that may kill them before reproduction can occur. Accordingly, one should expect a time threshold, when oocytes are sufficiently mature to be released, from when females’ interests should turn to favor oocyte immune protection as a terminal investment before their death. 

In a previous study, we hypothesized that worms from the most polluted site of Boulogne might have evolved some tolerance mechanisms to pollution, showing some immune parameters less affected and higher survival to experimental infection compared to worms from Gravelines, whose contact with pollution is more recent [31]. We wondered if such a tolerance mechanism could be expressed in the context of TGIP. We did observe a tendency of females from Boulogne to transfer more hedistin mRNA after saline injection. However, when injected with bacteria, females from Boulogne appeared as inefficient as those from Gravelines to transfer immune protection to their oocytes. Interestingly, these females tended nevertheless to accumulate more MPII mRNA in their oocyte after a bacterial challenge, but this result would need to be checked, e.g., with a larger sample size. MPII are small cadmium-binding proteins that are produced by a population of immune cells of *H. diversicolor* (G1) and by intestinal cells. They have bactericidal properties but are also proposed to be involved in metal detoxification by trapping the excess of essential or non-essential free metal ions [59]. If it is confirmed, such maternal transfer may be less costly for tolerant females of Boulogne, may help future embryos to better cope with simultaneous multiple stress, despite the inability of the mothers to enhance their immune investment in their progeny.

Overall, many authors agreed to consider that transgenerational plasticity could play a major role in species evolution, especially in changing environments [60]. The consequences of life in the polluted zone on TGIP efficiency highlighted here suggested that the effect of environmental contaminants on local populations might be deeper than previously thought. This deserves further investigation to better apprehend future biodiversity in a largely contaminated world.

## Figures and Tables

**Table 1:** Kruskal–Wallis (KW) tests.

Sample Type	Antibacterial Activity Tested	KW Chi-Squared	df	*p*-Value
Plasma	Anti-bacillus	15.35	3	0 *
Oocytes	Anti-bacillus	11.94	3	0.01 *
Oocytes	Anti-vibrio	12.42	3	0.01 *

Statistically significant differences, detected at *p* ≤ 0.05, are highlighted with an asterisk (*). df, degree of freedom.

**Table 2:** Post hoc Dunn tests.

Comparison	z Satistic	Adjusted *p*-value
Plasma anti-bacillus activity		
control vs. saline	0.15	0.44
control vs. bacillus	3.34	0.0002 *
control vs. vibrio	2.20	0.028
saline vs. bacillus	2.96	0.005 *
saline vs. vibrio	−1.96	0.037
bacillus vs. vibrio	0.58	0.34
Oocyte anti-bacillus activity		
control vs. saline	0.26	0.47
control vs. bacillus	3.02	0.007 *
control vs. vibrio	−0.35	0.54
saline vs. bacillus	2.69	0.011 *
saline vs. vibrio	−0.08	0.47
bacillus vs. vibrio	2.67	0.008 *
Oocyte anti-vibrio activity		
control vs. saline	1.41	0.09
control vs. bacillus	3.05	0.007 *
control vs. vibrio	−2.95	0.005 *
saline vs. bacillus	1.57	0.12
saline vs vibrio	−1.47	0.11
bacillus vs. vibrio	0.10	0.46

Statistically significant differences, detected at *p* ≤ 0.025 after Benjamini–Hochberg adjustment for multiple comparisons, are highlighted with an asterisk (*).

**Table 3:** Permutational analyses of variance for each investigated gene.

Tested Effect	df	R Sum of Square	R Mean Square	nb of Iterations	*p*-Value
Hedistin					
Treatment	2	4.3503	2.1751	5000	0.016 *
Site	2	6.8946	3.4473	5000	0.0026 *
treatment:site	4	8.9560	2.2390	5000	0.012 *
Residuals	27	14.7338	0.5457		
Lysozyme					
Treatment	2	0.002536	0.00126792	51	0.84
Site	2	0.000438	0.00021904	69	0.72
treatment:site	4	0.003470	0.00086757	232	0.85
Residuals	27	0.046668	0.00172844		
MPII					
Treatment	2	18.112	9.0561	5000	0.042 *
Site	2	4.350	2.1752	260	0.55
treatment:site	4	12.455	3.1136	680	0.37
Residuals	27	77.318	2.8636		

Statistically significant differences, detected at *p* ≤ 0.05, are highlighted with an asterisk (*). MPII, metalloprotease II.

## Supplementary Materials

The following are available online at https://www.mdpi.com/2073-4425/10/12/989/s1, S1: supporting datasets.

## Appendix A

**Global model for hedistin**:

Hedistin <- lmp (Hed_ratio~treatment*site, data = ovo_ratio, perm = "Exact", seqs = TRUE, center = TRUE)

Table A1 Results of Tukey multiple comparisons of means for Hedistin gene expression.

**Table:** $treatment

	Diff	lwr	upr	p adj
SS-C	0.70964750	−0.03808968	1.4573847	0.0653320
B-C	0.76235583	0.01461865	1.5100930	**0.0450242**
B-SS	0.05270833	−0.69502885	0.8004455	0.9833114

**Table:** $site

	Diff	lwr	upr	p adj
GR-AN	−1.0318808	−1.77961801	−0.2841437	**0.0054865**
BO-AN	−0.2644583	−1.01219551	0.4832788	0.6591841
BO-GR	0.7674225	0.01968532	1.5151597	**0.0434061**

**Table:** $‘treatment:site’

	Diff	lwr	upr	p adj
SS:AN-C:AN	1.0761000	−0.68144271	2.83364271	0.5184219
B:AN-C:AN	2.0714000	0.31385729	3.82894271	**0.0121837**
C:GR-C:AN	−0.2158675	−1.97341021	1.54167521	0.9999670
SS:GR-C:AN	−0.1230500	−1.88059271	1.63449271	0.9999996
B:GR-C:AN	0.3907750	−1.36676771	2.14831771	0.9974183
C:BO-C:AN	0.5950250	−1.16251771	2.35256771	0.9623583
SS:BO-C:AN	1.5550500	−0.20249271	3.31259271	0.1142269
B:BO-C:AN	0.2040500	−1.55349271	1.96159271	0.9999785
B:AN-SS:AN	0.9953000	−0.76224271	2.75284271	0.6160078
C:GR-SS:AN	−1.2919675	−3.04951021	0.46557521	0.2878486
SS:GR-SS:AN	−1.1991500	−2.95669271	0.55839271	0.3787317
B:GR-SS:AN	−0.6853250	−2.44286771	1.07221771	0.9190025
C:BO-SS:AN	−-0.4810750	−2.23861771	1.27646771	0.9896603
SS:BO-SS:AN	0.4789500	−1.27859271	2.23649271	0.9899499
B:BO-SS:AN	−0.8720500	−2.62959271	0.88549271	0.7587755
C:GR-B:AN	−2.2872675	−4.04481021	−0.52972479	**0.0043480**
SS:GR-B:AN	−2.1944500	−3.95199271	−0.43690729	**0.0068008**
B:GR-B:AN	−1.6806250	−3.43816771	0.07691771	0.0690933
C:BO-B:AN	−1.4763750	−3.23391771	0.28116771	0.1536504
SS:BO-B:AN	−0.5163500	−2.27389271	1.24119271	0.9838428
B:BO-B:AN	−1.8673500	−3.62489271	−0.10980729	**0.0309716**
SS:GR-C:GR	0.0928175	−1.66472521	1.85036021	1.0000000
B:GR-C:GR	0.6066425	−1.15090021	2.36418521	0.9579946
C:BO-C:GR	0.8108925	−0.94665021	2.56843521	0.8208871
SS:BO-C:GR	1.7709175	0.01337479	3.52846021	**0.0472151**
B:BO-C:GR	0.4199175	−1.33762521	2.17746021	0.9957792
B:GR-SS:GR	0.5138250	−1.24371771	2.27136771	0.9843272
C:BO-SS:GR	0.7180750	−1.03946771	2.47561771	0.8976455
SS:BO-SS:GR	1.6781000	−0.07944271	3.43564271	0.0698178
B:BO-SS:GR	0.3271000	−1.43044271	2.08464271	0.9992660
C:BO-B:GR	0.2042500	−1.55329271	1.96179271	0.9999784
SS:BO-B:GR	1.1642750	−0.59326771	2.92181771	0.4164727
B:BO-B:GR	−0.1867250	−1.94426771	1.57081771	0.9999892
SS:BO-C:BO	0.9600250	−0.79751771	2.71756771	0.6583542
B:BO-C:BO	−0.3909750	−2.14851771	1.36656771	0.9974091
B:BO-SS:BO	−1.3510000	−3.10854271	0.40654271	0.2381011

**Global model for lysozyme**:

Lysozyme <- lmp(Lys_ratio~treatment*site, data = ovo_ratio, perm = "Exact", seqs = TRUE, center = TRUE)

**Global model for MPII**:

MPII <- lmp(MPII_ratio~treatment*site, data = ovo_ratio, perm = "Exact", seqs = TRUE, center = TRUE)

Table A2 Results of Tukey multiple comparisons of means for MPII gene expression.

**Table:** $treatment

	Diff	lwr	upr	p adj
SS-C	0.527450	−1.18544805	2.240348	0.7281813
B-C	1.697383	−0.01551471	3.410281	0.0524671
B-SS	1.169933	−0.54296471	2.882831	0.2259946

**Table:** $site

	Diff	lwr	upr	p adj
GR-AN	−0.7890167	−2.501915	0.9238814	0.4971996
BO-AN	−0.6718000	−2.384698	1.0410980	0.6001904
BO-GR	0.1172167	−1.595681	1.8301147	0.9842637

**Table:** $‘treatment:site’

	Diff	lwr	upr	p adj
SS:AN-C:AN	1.28700	−2.7391359	5.313136	0.9730468
B:AN-C:AN	1.22725	−2.7988859	5.253386	0.9797379
C:GR-C:AN	−0.21955	−4.2456859	3.806586	0.9999999
SS:GR-C:AN	−0.11375	−4.1398859	3.912386	1.0000000
B:GR-C:AN	0.48050	−3.5456359	4.506636	0.9999735
C:BO-C:AN	−0.95185	−4.9779859	3.074286	0.9960699
SS:BO-C:AN	−0.76230	−4.7884359	3.263836	0.9991693
B:BO-C:AN	2.21300	−1.8131359	6.239136	0.6511734
B:AN-SS:AN	−0.05975	−4.0858859	3.966386	1.0000000
C:GR-SS:AN	−1.50655	−5.5326859	2.519586	0.9346584
SS:GR-SS:AN	−1.40075	−5.4268859	2.625386	0.9560796
B:GR-SS:AN	−0.80650	−4.8326359	3.219636	0.9987572
C:BO-SS:AN	−2.23885	−6.2649859	1.787286	0.6376578
SS:BO-SS:AN	−2.04930	−6.0754359	1.976836	0.7340597
B:BO-SS:AN	0.92600	−3.1001359	4.952136	0.9967418
C:GR-B:AN	−1.44680	−5.4729359	2.579336	0.9474613
SS:GR-B:AN	−1.34100	−5.3671359	2.685136	0.9657253
B:GR-B:AN	−0.74675	−4.7728859	3.279386	0.9992840
C:BO-B:AN	−2.17910	−6.2052359	1.847036	0.6687668
SS:BO-B:AN	−1.98955	−6.0156859	2.036586	0.7625830
B:BO-B:AN	0.98575	−3.0403859	5.011886	0.9950245
SS:GR-C:GR	0.10580	−3.9203359	4.131936	1.0000000
B:GR-C:GR	0.70005	−3.3260859	4.726186	0.9995522
C:BO-C:GR	−0.73230	−4.7584359	3.293836	0.9993784
SS:BO-C:GR	−0.54275	−4.5688859	3.483386	0.9999329
B:BO-C:GR	2.43255	−1.5935859	6.458686	0.5354583
B:GR-SS:GR	0.59425	−3.4318859	4.620386	0.9998670
C:BO-SS:GR	−0.83810	−4.8642359	3.188036	0.9983693
SS:BO-SS:GR	−0.64855	−4.6746859	3.377586	0.9997448
B:BO-SS:GR	2.32675	−1.6993859	6.352886	0.5913068
C:BO-B:GR	−1.43235	−5.4584859	2.593786	0.9502803
SS:BO-B:GR	−1.24280	−5.2689359	2.783336	0.9781308
B:BO-B:GR	1.73250	−2.2936359	5.758636	0.8688633
SS:BO-C:BO	0.18955	−3.8365859	4.215686	1.0000000
B:BO-C:BO	3.16485	−0.8612859	7.190986	0.2148936
B:BO-SS:BO	2.97530	−1.0508359	7.001436	0.2817444
